# Long‐term hippocampal alterations and cognitive impairment in a murine model of surgical sepsis

**DOI:** 10.1002/2211-5463.70240

**Published:** 2026-04-16

**Authors:** Dong Seong Cho, Rebecca Schmitt, Aneesha Dasgupta, Alexandra Ducharme, Jason Doles

**Affiliations:** ^1^ Department of Biochemistry and Molecular Biology Mayo Clinic Rochester Minnesota USA; ^2^ Department of Anatomy, Cell Biology, and Physiology Indiana University School of Medicine Indianapolis USA; ^3^ Indiana Center for Musculoskeletal Health Indianapolis USA

**Keywords:** brain, cecal ligation and puncture (CLP), memory, sepsis‐associated brain dysfunction (SABD), single‐cell RNA sequencing

## Abstract

Cognitive impairment in sepsis survivors is a growing clinical challenge as the number of sepsis cases rise and acute mortality rates fall. It is estimated that up to 70% of sepsis patients experience cognitive dysfunction and many report persistent dysfunction post‐sepsis. Cognitive impairment is a broad term that refers to sepsis‐associated brain dysfunction or encephalopathy (SABD or SAE, respectively). While many contributors, such as inflammation, blood–brain barrier dysfunction, and extended microglial activation are implicated in SABD/SAE development, much is still unknown regarding the underlying mechanistic causes of persistent cognitive defects in sepsis survivors. Here, we employed cecal ligation and puncture (CLP) to model surgical sepsis in mice, after which mice were subjected to multiple memory function assays for up to 2 months post‐sepsis. Furthermore, single‐cell RNA sequencing and validation studies were performed with murine hippocampi to query alterations in the brain microenvironment. Our study shows that CLP mice exhibited decreased spontaneous alternation at 4 weeks and decreases in both short‐ and long‐term recognition (at 6 weeks and 2 weeks, respectively) accompanied by substantially altered cell type makeup of the hippocampus, including hippocampal neuron and pericyte loss. Finally, transcriptional alterations in the macrophage populations implicate aberrant activation in CLP survivors. In conclusion, post‐sepsis mice exhibit reduced memory capacity and altered hippocampal cell composition and transcriptional profiles. This study and associated data set will be valuable for further investigation of the underlying pathophysiology of SABD/SAE and elucidation of potential therapeutic candidates.

## Graphical abstract

Using a mouse model of surgical sepsis, we tested long‐term memory and analyzed the transcriptome of single cells isolated from the hippocampus. Survivor mice showed worse memory, loss of certain brain cell subpopulations, and abnormal immune cell activity—suggesting that post‐sepsis brain alterations may be linked to cognitive deficits. Image created with BioRender.

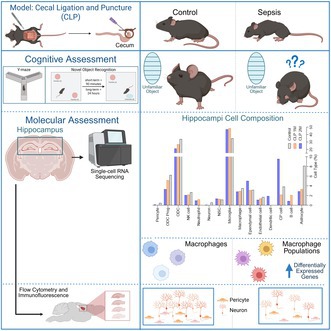

**Abbreviations**
1 M: one month
2 M: two month
ANOVA: analysis of variance
BMC: Bonferroni Multiple Comparisons; standard error of meanSEM)
BSA: bovine serum albumin
CCI: chronic critical illness
CLP: cecal ligation and puncture
CO_2_
: carbon dioxide
CP: choroid plexus
DAMPs: damage‐associated molecular pathways
DEGs: differentially expressed genes
EDTA: ethylenediaminetetraacetic acid
FACS: fluorescence‐activated cell sorting
FcR: Fc receptor
ICU: intensive care unit
IPA: Ingenuity Pathway Analysis
LPS: lipopolysaccharide
M0: macrophagesnon‐polarized
M1: macrophages pro‐inflammatory
M2: macrophages anti‐inflammatory
NeuN: neuronal nuclear protein
NK: natural killer cells
NOS2: inducible nitric oxide synthase
NSC: neural stem cells
OCT: optimal cutting temperature
ODC Prog: oligodendrocyte progenitor cells
ODC: oligodendrocytes
PBS: phosphate‐buffered saline
PCI: peritoneal contamination and infection
RNA: ribonucleic acid
SABD: Sepsis‐associated brain dysfunction
SAE: sepsis‐associated encephalopathy
sAKI: sepsis‐associated acute kidney injury
scRNAseq: single‐cell RNA sequencing
UMAP: uniform manifold approximation and projection

Sepsis, defined by Singer et al as a dysregulated host response to infection that causes life‐threatening organ dysfunction [1], is a major contributor (~ 20%) to the global death rate [2]. Despite this sobering statistic, sepsis mortality rates are falling as identification and treatment of septic shock improve. This is leading to a growing population of sepsis survivors, among whom approximately 50% experience chronic critical illness (CCI) [3]. CCI is a broad term and encompasses diverse sepsis sequelae including (but not limited to) cognitive impairment, organ dysfunction, muscle wasting, and increased infection risk [3, 4].

Brain/cognitive dysfunction caused by sepsis, termed sepsis‐associated encephalopathy (SAE), or more recently sepsis‐associated brain dysfunction (SABD), is not well understood but is thought to affect between ~ 20–70% of sepsis patients [5, 6, 7, 8, 9]. Many factors contribute to this highly variable estimate and include (i) difficulty in assessing neurological function/alterations in patients on certain treatments (e.g., sedatives, opioids), (ii) vague diagnostic criteria (i.e., based on exclusion), and (iii) a highly complex underlying etiology [9, 10]. Clinically, SABD is characterized by delirium (reduced rationality/awareness), coma, mental processing, mood disorders, cognitive/memory dysfunction, and more. These diverse phenotypes are seen acutely in the ICU and can persist long‐term [10, 11, 12]. Severe sepsis survivors have a 3.3‐fold increase in the development of moderate/severe cognitive impairments compared to non‐sepsis hospitalization/ICU patients [13]. Further, sepsis patients that exhibit acute “altered mental status” have mortality rates nearly double those exhibiting “normal mental status” (49% versus 26%, respectively) [5].

Rodent and human studies have identified several mechanistic contributors to SAE/SABD including hippocampus alterations [14, 15], neuronal cell loss [16], increased permeability/disruption of the blood–brain barrier [17, 18], and inflammation and microglial activation [19, 20]. While these studies have been impactful, we still do not understand the full scope of the changes occurring in the brain that cause long‐term dysfunction. In the present study, we sought to define transcriptional and cell type changes in the hippocampus with the goal of better understanding mechanisms of post‐sepsis cognitive dysfunction. Using a gold‐standard murine sepsis model, we performed longitudinal working memory and short−/long‐term memory function tests to assess post‐sepsis cognitive deficits. Subsequently, hippocampi were isolated from control, 1 month, and 2 months post‐septic mice and analyzed via single‐cell RNA sequencing (scRNAseq). We observed significant reductions in neuron and pericyte abundance while differential gene expression analysis revealed persistent activation of selected macrophage populations at both 1‐ and 2‐months post‐sepsis. We anticipate that this study will serve as a valuable resource for those interested in studying sepsis recovery and molecular/cellular drivers of long‐term post‐sepsis cognitive dysfunction.

## Materials and methods

### Animals and CLP procedure

All procedures using animals were performed at Mayo Clinic (Rochester, MN; PHS Assurance ID D16‐00187) or at Indiana University School of Medicine/Indiana University Indianapolis (Indianapolis, IN; PHS Assurance ID D16‐00584) and were approved by Institutional Animal Care and Use Committees. 14‐month‐old female C57Bl/6 mice were purchased from Jackson Laboratory (catalog #000664) and were acclimated for two weeks before use. All mice were housed according to NIH guidelines in a pathogen‐free facility. The cecal ligation and puncture (CLP) model was performed as previously described [21]. Briefly, mice were first randomized between groups (control and CLP), and then the CLP groups were anesthetized with isoflurane (Pirmal), and cecum was extracted from the abdomen and ligated approximately 1.0 cm from the tip. A single hole was punctured in the cecum between the tip and ligation with a 25‐G needle (BD Biosciences), fecal matter extruded, and replaced in the abdomen. Prior to surgery, mice received subcutaneous dosages of 0.5‐mg·kg^−1^ buprenorphine SR‐LAB (ZooPharm–0.5 mg·mL^−1^) and 4 mg·kg^−1^ Meloxicam SR (ZooPharm–2 mg·mL^−1^). Post‐operation, mice received a 1‐mL subcutaneous injection of warmed saline (BD Biosciences) for resuscitation. CLP mice received intraperitoneal injections of antibiotics (25 mg·kg^−1^ imipenem monohydrate (LKT Labs or Med Chem Express)) post‐surgery and twice daily for the next 72 h. Criteria for humane endpoint were as follows: hunched posture (score 0–2), ruffled coat (score 0–2), diarrhea (score 0–2), dehydrated eyes (score 0–2), reduced temperature (score 0–1), and reluctance to move (score 0–2) where a cumulative clinical score of 6 or above will necessitate humane euthanasia. No exclusion criteria were used. Euthanasia was performed using CO_2_ inhalation followed by secondary physical methods in accordance with institutional protocols.

### Cognitive behavior assessments

Y‐maze test was performed as described in previous studies [22, 23]. Briefly, a mouse was positioned within one arm of a Y‐shaped maze, facing the endpoint wall. The sequence and number of arm entries were recorded and tracked for 5 min using a video‐tracking system, EthoVision XT (Noldus Information Technology, VA, USA). Spatial working memory was quantified and evaluated with a spontaneous alternation (%). Spontaneous alternation is defined as the number of alternations divided by the number of total sequent three‐arm entries and multiplied by 100: spontaneous alternation (%) = [(the number of alternations)/(total arm entries−2)] × 100.

To evaluate short‐term and long‐term memory, the novel object recognition test was performed as described previously [23]. Briefly, a mouse was first habituated to an open field chamber (40 × 40 × 30 cm) for 5 min. The mouse was then placed in the open field chamber with two identical objects, which were positioned in two adjacent corners, 9 cm from the walls. The mouse was allowed to explore the objects for 5 min of a training session and then returned to its cage. For the memory tests, one of the two objects in the open field chamber was replaced by a novel object. For short‐term memory, mice were placed back into the open field 90 min after the initial training session, whereas for long‐term memory it was 24 h after the initial training session. Exploration of the objects was recorded and tracked using EthoVision XT (Noldus Information Technology, VA, USA). A novel object recognition index was calculated for each mouse as follows: [(time spent exploring the novel object)/(total time spent exploring all two objects)] × 100. Between each session of animals, the objects and the open field chamber were cleaned with 70% ethanol. All behavioral assessments were conducted under blinding whenever possible.

### Murine hippocampus single‐cell RNA sequencing (scRNAseq)

Mouse hippocampus was dissected, and three mice from each condition were pooled to generate a single replicate (*n* = 1) for sequencing. Single cells from the hippocampi were dissociated and prepared using the Adult Brain Dissociation Kit (Miltenyi Biotec, Germany) according to the manufacturer's protocol. In brief, dissociated cells were filtered through a 70 μm cell strainer and mixed with propidium iodide solution (1 : 100) (Miltenyi Biotec, Germany). Propidium iodide negative populations (live cells) were sorted by fluorescence‐activated cell sorting (FACS) at the Mayo Clinic Microscopy Cell Analysis Core Flow Cytometry Facility (Rochester, MN, USA).

ScRNAseq and associated analyses were performed as previously described [21], with several modifications. Freshly isolated single cells were captured and complementary DNA libraries prepared using Chromium Single Cell Reagent Kit v2 (10× Genomics, CA, USA). Sequencing was performed using a HiSeq4000 with paired‐end 100 bp reads. Sequencing data was processed and aligned to the mm10 genome using Cell Ranger 3.1.0 (10× Genomics, CA, USA). Data were analyzed with Seurat package (version 2.3.4) [24, 25] using RStudio software. Cells with poor sequencing quality were excluded from analysis by removing cells with high fraction of mitochondrial transcripts (> 10%), with low unique molecular identifier counts (< 2000), or with low number of detected genes (< 1000). Markers to classify each cluster into a specific cell type based on PanglaoDB database [26], shown in Figs S1, S2. Differentially expressed genes (DEGs) in each cell type were found by FindMarkers function in Seurat package with the following thresholds (fold change > 1.5, *P* < 0.01, and adjusted *P* < 0.05). DEGs were used for pathway analysis using Ingenuity Pathway Analysis software (QIAGEN Inc., Germany, https://www.qiagenbioinformatics.com/products/ingenuitypathway‐analysis).

### Immunohistochemistry

Brain tissue was fixed and dissected with whole animal perfusion fixation as described previously [27]. After fixation in 4% paraformaldehyde in phosphate‐buffered saline (PBS), brain preparations were incubated in PBS with 30% (w/v) sucrose for 3 days at 4 °C. After freezing fixed and dehydrated brains using dry ice, brain samples were embedded in OCT and sectioned at 40 μm. Tissue sections were permeabilized in PBS with 0.2% Triton X‐100 and 5% bovine serum albumin (BSA) at room temperature for 1 h. Tissues were incubated with primary antibodies in PBS with 5% BSA overnight at 4 °C, followed by incubation with secondary antibodies at room temperature for 1 h and 40 min. The following antibodies were used in this study at the indicated dilutions: NeuN antibody (1 : 100) (Novus Biologicals, NBP1‐77686), CD13 antibody (1 : 100) (Bio‐Rad, MCA2183), Alexa Fluor 647‐conjugated goat anti‐rat secondary antibody (1 : 500) (Thermo Fisher, A‐21247), and Alexa Fluor 488‐conjugated goat anti‐rabbit secondary antibody (1 : 500) (Thermo Fisher, A‐11008). Tissue sections were then incubated with 1 μg·mL^−1^ DAPI for 15 min.

### Flow cytometry

Freshly isolated brain cells (see above for scRNA seq studies) were fixed in 4% paraformaldehyde in PBS at room temperature for 10 min, followed by incubation in PBS with 0.2% Triton X‐100 and 5% BSA at room temperature for 10 min. Cells were then incubated with NeuN antibody (1 : 10) (Novus Biologicals, NBP1‐77686) and FcR blocking reagent (1 : 10) (Miltenyi Biotec, Germany) at room temperature for 10 min, followed by incubation with Alexa Fluor 488 conjugated goat anti‐rabbit secondary antibody (1 : 60) (Thermo Fisher, A‐11008) at room temperature for 10 min. Cells were spun down and then resuspended in PBS supplemented with 2 mm EDTA, 0.5% BSA, and propidium iodide solution (1 : 100) (Miltenyi Biotec, Germany). Flow cytometry analysis was performed using a MACSQuant instrument (Miltenyi Biotec, Germany).

### Statistical analyses

GraphPad Prism versions 9 and 10 (GraphPad Software, Boston, MA, www.graphpad.com) for windows was used to perform all statistical analyses. Comparisons with two groups were performed with Student's *t*‐test with Welch's correction. Three or more group comparisons were analyzed with one‐way ANOVA with Bonferroni Multiple Comparisons (BMC).

## Results

### Cognitive alterations in sepsis survivor mice

**Fig. 1:**
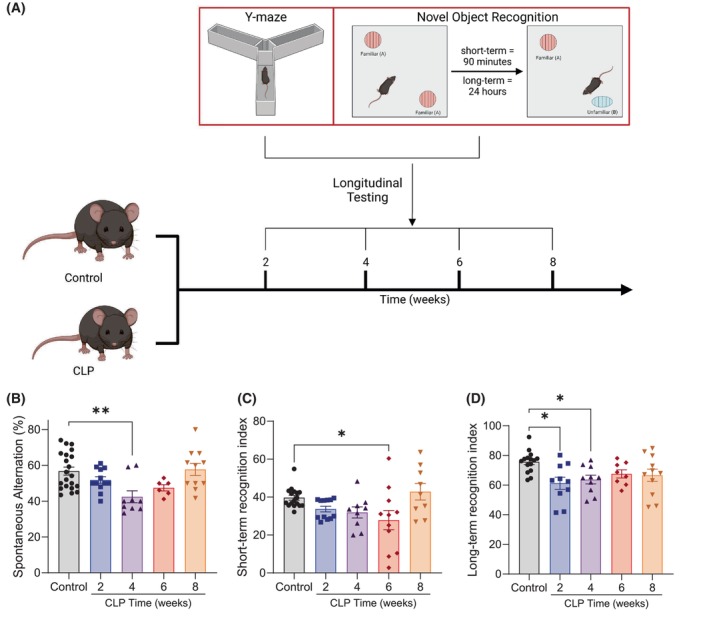
Impaired memory function in sepsis survivor mice. (A) Schematic depicting timeline and memory tests performed on control and cecal ligation and puncture (CLP) mice. Created with BioRender. (B) Quantification of spontaneous alternation from Y‐maze in control and CLP mice at 2, 4, 6, and 8 weeks. (C) Short‐term recognition index from the 90‐min novel object recognition test in CLP mice at 2, 4, 6, and 8 weeks and control mice. (D) Control and CLP mice at 2‐, 4‐, 6‐, and 8‐weeks long‐term recognition index from 24‐h novel object recognition test. B–D = One‐way ANOVA with Bonferroni multiple comparisons (BMC). Data is represented as mean ± SEM. Significance: *P < 0.05, **P < 0.01, ***P < 0.001. B, control *n* = 21, CLP 2w *n* = 12, CLP 4w *n* = 9, CLP 6w *n* = 6, and CLP 8w *n* = 11. C, control *n* = 17, CLP 2w *n* = 12, CLP 4w *n* = 9, CLP 6w *n* = 11, and CLP 8w *n* = 9. D, control *n* = 17, CLP 2w *n* = 12, CLP 4w *n* = 12, CLP 6w *n* = 10, and CLP 8w *n* = 13.

The first objective of this study was to determine the extent of cognitive impairment in mice surviving cecal ligation and puncture (CLP). We chose to use middle‐aged mice because (i) most patients diagnosed with sepsis are middle‐aged and older, and (ii) this population is most affected by post‐sepsis cognitive impairment. Control (no CLP surgery; see methods) and post‐CLP mice underwent Y‐maze and novel object recognition testing at 2‐, 4‐, 6‐, and 8‐weeks post‐CLP surgery to measure spontaneous alternation/working memory and short‐ and long‐term recognition, respectively (Fig. 1A). Although CLP mice at 2‐, 4‐, and 6‐weeks post‐surgery exhibited a slight decrease in spontaneous alternation, only the 4‐week timepoint was significantly decreased (~14% reduction) compared to control (Fig. 1B). Further, while CLP mice had a significant decrease in total distance traveled and number of arm entries (Fig. S3a,b, respectively) in the Y‐maze, there was no correlation between spontaneous alternation and total distance (Fig. S3c) or number of arm entries (Fig. S3d).

Similar to the spontaneous alternation results, 2‐, 4‐, and 6‐weeks post‐CLP mice did not exhibit major changes in short‐term recognition, with the only significant time point being a ~ 30% loss in the short‐term recognition index at the 6‐week time point compared to controls (Fig. 1C). Conversely, post‐CLP mice had a significant deficit in long‐term memory at 2‐ and 4‐weeks post‐CLP by ~ 17% and 15%, respectively, versus controls (Fig. 1D). For all memory tests, complete functional recovery was observed by 8 weeks post‐CLP.

### CLP‐induced cell composition changes in the murine brain

**Fig. 2:**
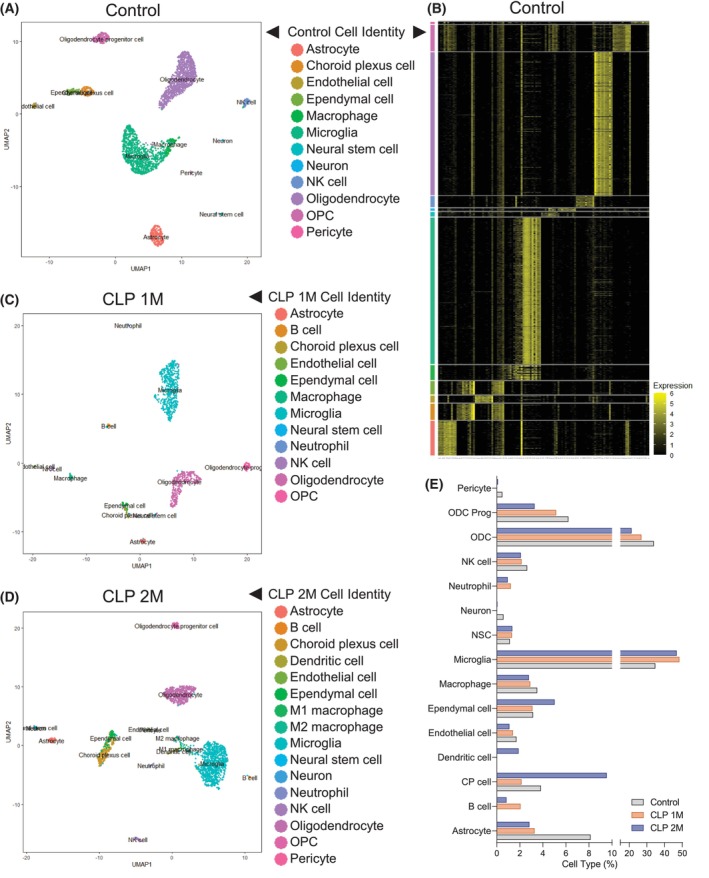
Single‐cell RNA sequencing cell type identification in control and post‐CLP mouse hippocampi. (A) Uniform manifold approximation and projection (UMAP) depicting identified cell type clusters from control mice hippocampus. (B) Heatmap depicting control hippocampal top differentially expressed genes (DEGs) per identified cell type cluster. Yellow: higher expression and black: lower expression. (C, D) UMAP depiction of identified cell types in mouse hippocampus at 1‐month (CLP 1 M; C) and 2‐months post‐CLP (CLP 2 M; D). (E) Cell type percentage comparison across CLP 1 M and CLP 2 M. NK, natural killer cell; OPC, oligodendrocyte progenitor cell; ODC, oligodendrocyte; NSC, neural stem cell; CP, choroid plexus. Each condition represents cells obtained from a single pooled (three murine hippocampi) sequencing replicate.

In an effort to begin understanding cellular and molecular alterations associated with observed cognitive deficits in post‐septic mice, we performed transcriptomics analyses [21] using single cells isolated from mouse hippocampi. After quality control, single cell data underwent dimensionality reduction via uniform manifold approximation and projection (UMAP). UMAP identified 20 different cell clusters (Fig. S4) that were categorized into specific cell types with cell type‐specific markers as reported in PanglaoDB database [26]. Twelve distinct cell types were identified (Figs 2A,B, S1, S2). Next, hippocampi from mice at 1‐ and 2‐months post‐CLP (CLP 1 M and CLP 2 M, respectively) were processed for scRNAseq. Following the same pipeline as control cells, CLP 1 M and CLP 2 M cells underwent cluster/maker analysis. Individual UMAP projections from CLP 1 M and 2 M demonstrate altered hippocampal cell type abundance in mice at both experimental timepoints (Fig. 2C,D). Cell type percentages were calculated and used to compare relative abundance across the three samples (Fig. 2E). We identified progressive decreases (control– > CLP 1 M– > CLP 2 M) in abundance of the following cell types: oligodendrocyte progenitor cells (ODC Prog), oligodendrocytes (ODC), and astrocytes. Interestingly, some cell types were identified in the CLP cohorts that were not present at appreciable levels/identified in the control samples including neutrophils, dendritic cells, and B‐cells. Notably, pericyte and neuron populations were nearly absent in both CLP timepoints. These data suggest that the cell composition of the mouse hippocampi is significantly altered by CLP, with only a few cell types not overtly affected in terms of relative abundance (e.g., natural killer cells (NK), neural stem cell (NSC), macrophages, and endothelial cells).

**Fig. 3:**
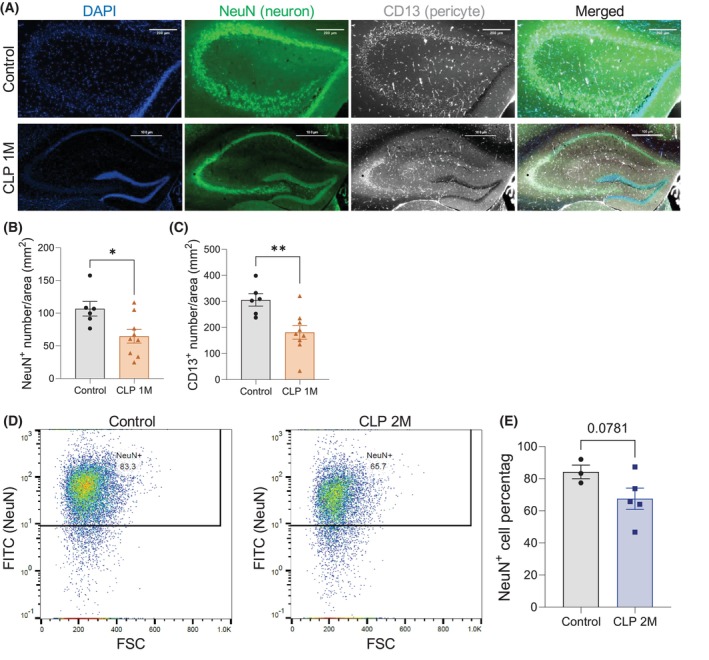
Loss of neuronal and pericyte cell populations in sepsis survivor mice. (A) Immunofluorescence (IF) of control and CLP 1 M hippocampus sections. Blue = DAPI; Green = neuronal nuclear protein (NeuN); White = pericyte marker (CD13). Images are representative and taken at x10, scale bars: 200 μm. (B, C) Quantification of NeuN (B) and CD13 (C) positive cells per area. (D) Flow cytometry analysis of control and CLP 2 M hippocampus samples incubated with NeuN. (E) Quantified values of NeuN‐positive cells in control and CLP 2 M. B, C and E = Student's unpaired t‐test with Welch's correction. Data is represented as mean ± SEM. Significance: *P < 0.05, **P < 0.01, ***P < 0.001. B and C, control *n* = 6 and CLP 1 M *n* = 9; E, control *n* = 3 and CLP 2 M *n* = 5.

Given the prominent reduction of neurons and pericytes in both CLP 1 M and 2 M cohorts in our scRNAseq data, we next sought to validate these results using independent samples and orthogonal techniques. CLP 1 M and control brains were sectioned and hippocampal regions assessed using antibodies recognizing neuronal nuclear protein (NeuN), a marker for neurons, and CD13, a pericyte marker (Fig. 3A). Quantification of NeuN‐positive cells revealed a significant decrease in NeuN signal in CLP 1 M hippocampi compared to controls (Fig. 3B). Similar results were obtained when quantifying CD13‐positive cells per area, where CLP 1 M also had reduced numbers of CD13+ cells compared to controls (Fig. 3C). We further utilized flow cytometry to quantify the percentage of NeuN‐positive cells harvested from control and CLP 2 M hippocampi (Fig. 3D). Flow analysis revealed a trending decrease (p = 0.078) of NeuN+ cells in CLP 2 M compared to controls (Fig. 3E). Thus, while not as striking as the computed cell population reductions, these data support the validity of the scRNAseq data as a tool to gain insights into post‐CLP hippocampal remodeling.

### Differential gene expression analyses implicate chronic activation of multiple macrophage populations in the post‐CLP brain

**Fig. 4:**
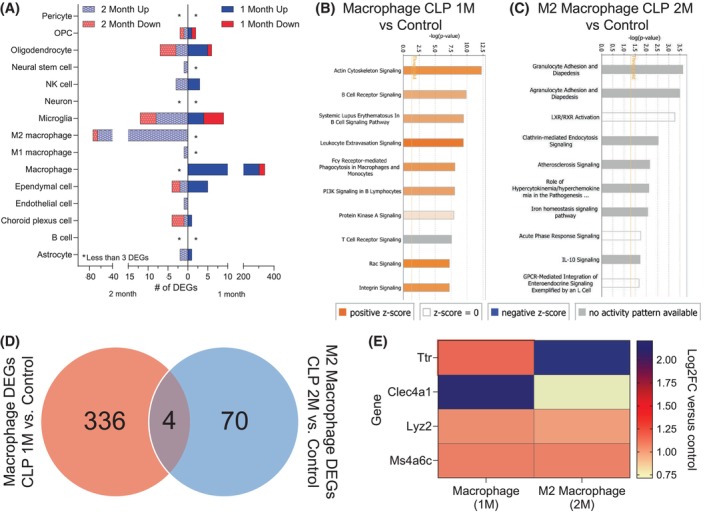
Differential gene expression analyses implicate prolonged macrophage activation in the post‐sepsis brain. (A) Number of differentially expressed genes (DEGs) that are upregulated (blue) or downregulated (red) in different hippocampal cell types at 1 month (solid color bars) and 2 months (dotted color bars) post‐CLP. *Indicates less than three DEGs. (B, C) Ingenuity Pathway Analysis of macrophage cell population at 1 month post‐CLP versus control (B) and M2 macrophage cell populations at 2 months post‐CLP versus control (C). (D) A Venn diagram depicting overlap between CLP 1 M and CLP 2 M DEGs. (E) A heatmap depicting the four overlapping DEGs between CLP 1 M and CLP 2 M timepoints.

As a next step, we sought to assess differentially expressed genes (DEGs) within each identified cell type in our control and CLP mice (Fig. 4A). Most cell types had a predominance of induced DEGs (blue bars) compared to decreased DEGs (red bars), and we did not observe any clear DEG abundance patterns between CLP 1 M and 2 M samples (each compared to control). We decided to look further into macrophage populations as previous studies highlighted the importance of microglia (brain resident macrophages) in SAE/SABD [19, 28]. Although the overall abundance of total macrophages did not appear to substantially change across samples (Fig. 2E), there were clear changes in non‐polarized (M0) macrophage, M1 macrophage, and M2 macrophage categorized cells (Fig. 4A). Macrophages in the CLP 1 M group had the highest number of DEGs (304 DEGs) in comparison to both CLP time points and across all cell types. Within CLP 2 M clusters, the M2 macrophage cell population had the highest amount of DEGs (66 DEGs). Ingenuity pathway analyses (IPA) of these DEGs from CLP 1 M macrophages and CLP 2 M M2 macrophages (Fig. 4B,C, respectively) point to activated states of these cell types—transcriptional signatures aligned with previous literature documenting microglia activation in response to sepsis‐associated stimuli [29]. Finally, we asked if there was any overlap between DEGs from these two post‐CLP populations (Fig. 4D) and found four overlapping transcripts: Ttr, Clec4a1, Lyz2, and Ms4a6c (Fig. 4E). Of these, three maintained the same directional change over time—Ttr, Lyz2, and Ms4a6c. Together, these data support the hypothesis that there is substantial immune remodeling and lasting immune activation/dysfunction in the post‐sepsis brain.

## Discussion

Understanding the molecular underpinnings that contribute to post‐sepsis cognitive impairment is important for improving patient quality of life and extending healthspan. Indeed, significant progress has been made towards understanding the etiology of sepsis‐associated brain dysfunction (SABD) and sepsis‐associated encephalopathy (SAE) [9, 11, 12, 30]. One shortcoming of many of these studies, however, is the emphasis on early/proximal post‐sepsis timepoints, which may not fully capture the lasting consequences of sepsis on the brain. Investigations that have extended their scope to longer‐term outcomes highlight both the persistence of neuropathological changes as well as the therapeutic potential of targeting SAE/SABD *in vivo*.

For example, Weberpals and colleagues demonstrated that inducible nitric oxide synthase (NOS2) protein expression is increased in wild‐type mouse hippocampus and frontal cortex up to 8 weeks after lipopolysaccharide (LPS) injection and that this elevation was associated with deficits in long‐term memory and increased numbers of activated microglia [19]. Loss of NOS2 using global knockout mice was able to preserve long‐term memory and partially rescue the activated microglia phenotype 8 weeks post LPS challenge [19]. Similarly, Chung and colleagues queried microglia and complement system activation in SAE [28]. They found that hippocampal microglia activate following post‐peritoneal contamination and infection (PCI). Specifically, microglia in post‐PCI mice exhibit increased expression of transcripts associated with phagocytosis and inflammation up to 20 days following PCI and this increased expression is accompanied by neuronal damage and decreased exploration of novel objects in novel object recognition tests administered 10‐ and 30 days post‐PCI [28]. Strikingly, C1q—a complement factor involved in tagging synapses for microglia engulfment—was significantly increased in PCI mouse hippocampi after 3 days [28]. Furthermore, increased co‐localization of C1q and synapses (via Homer1 staining) was found at 3‐ and 10 days post‐PCI, implicating synapse degradation [28]. When PCI mice were treated with the CSF1‐R (complement receptor found on microglia) inhibitor PLX5622 (PLX) starting 3 days post‐PCI to 10 days, treated mice had reduced microglia numbers, decreased expression of C1q in the hippocampus, and rescued time exploring novel objects at 10 and 30 days post‐PCI compared to PCI only mice [28]. Together, these studies showcase feasibility and the potential impact of targeting SAE/SABD to improve post‐sepsis cognitive outcomes, with the latter study further highlighting the efficacy of treatment post‐symptom onset which is an important practical consideration when thinking about clinical intervention.

In our own work, the highest upregulated transcript in both non‐polarized/M0 macrophages at CLP 1 M and M2 macrophages at CLP 2 M was Ttr. The consequence of altering Ttr in the hippocampus (or in different tissues) appears to be context dependent as Ttr can have deleterious or protective roles in disease or trauma. For example, Ttr was beneficial in a murine model of Alzheimer's disease [31] where the investigators found that TTR inhibition in the hippocampus (via an anti‐TTR antibody) led to increased *β*‐amyloid, neuronal loss, tau phosphorylation, and apoptosis. In this setting, TTR may be needed to help limit *β*‐amyloid accumulation and slow Alzheimer's disease progression [31]. In a second example, TTR increased neuron outgrowth in the hippocampus and provided neuroprotection in ischemic conditions [32]. Conversely, in a third study, overexpression of TTR in the mouse hippocampus was found to induce depression‐like behavior [33]. Fourth, while not in the brain, work to identify platelet‐derived damage‐associated molecular patterns (DAMPs) correlated with sepsis‐associated acute kidney injury (sAKI) implicated Ttr as one such candidate/target [34]. Here, Lv and colleagues used an *in vitro* co‐culture model whereby platelet produced TTR was able to induce reactive oxygen species and apoptosis in human proximal tubule epithelial cells, suggesting a negative role of Ttr with respect to the development of sAKI. Together, these studies highlight a key disease modifying role of Ttr and underscore the need to better define the role of Ttr in the post‐sepsis brain.

Taken together, our study leveraged a gold‐standard murine model of surgical sepsis (CLP) combined with cognitive testing and single cell transcriptomics to provide new insights into sepsis survivorship. We found that post‐sepsis mice exhibit (1) lasting deficits in memory/cognitive function, (2) evidence of neuron and pericyte loss in the hippocampus, and (3) significant and sustained cell‐type‐specific transcriptional changes, most notably in macrophage‐like populations. These data highlight enduring cellular molecular alterations that may underlie cognitive decline following sepsis.

Importantly, several limitations must be acknowledged. First, our analyses are primarily descriptive and thus cannot establish causality between the observed transcriptional changes and cognitive outcomes. Additionally, molecular and behavioral phenotype correlations are challenging due to the use of separate cohorts of mice (done so to avoid confounding effects of repeated testing on the transcriptome). Second, while we highlighted neuronal cell loss in the hippocampus, it is likely that other brain regions—as well as systemic factors—contribute substantially to SAE/SABD etiology. Indeed, there are likely multiple mechanisms contributing to the development of sepsis‐associated cognitive dysfunction and we acknowledge that follow‐up/validation studies are needed. Third, sepsis‐associated cognitive impairment likely evolves dynamically, thus requiring a more detailed longitudinal investigation than that captured by our limited number of experimental timepoints. Fourth, we acknowledge that this exploratory study was performed using only female mice. Indeed, there are known sex differences between males and females with respect to both acute sepsis severity as well as long‐term functional decline. Explicitly assessing sex differences post‐sepsis cognitive decline will be critical moving forward. Finally, although the CLP model remains a gold standard for experimental sepsis, its translational fidelity is inherently limited and should be complemented by investigations using human or large animal model systems.

Despite these caveats, our findings provide a valuable resource for the field and establish a foundation for hypothesis‐driven mechanistic studies. By querying behavioral, transcriptional, and cellular data, this work underscores the long‐lasting consequences of sepsis on brain health and supports the rationale for pursuing targeted interventions to mitigate cognitive decline in sepsis survivors.

## Conflict of interest

The authors declare no conflicts of interest.

## Author contributions

Study design: DSC and JDD. Data collection: DSC, RES, AD, and AMD. Data analysis/interpretation: DSC, RES, and JDD. Writing/editing of manuscript: RES and JDD.

## Supporting information


**Fig. S1.** Markers used for cell type identification.
**Fig. S2.** Markers used for cell type identification.
**Fig. S3.** Spontaneous alternation changes are not correlated with distance traveled or arm entries.
**Fig. S4.** Control hippocampus UMAP clustering.

## Data Availability

Data will be available at time of publication from the Sequence Read Archive (SRA): BioProject PRJNA1019387. All other data are available upon request.
