# Supplementary material for: Long‐term hippocampal alterations and cognitive impairment in a murine model of surgical sepsis
Source: FEBS Open Bio. 2026 Apr 16;16(9):1657–68. doi: 10.1002/2211-5463.70240 (PMC13398903; doi:10.1002/2211-5463.70240)
Supplement: Supplementary file 1 [file FEB4-16-1657-s001.pdf]

Figure S1

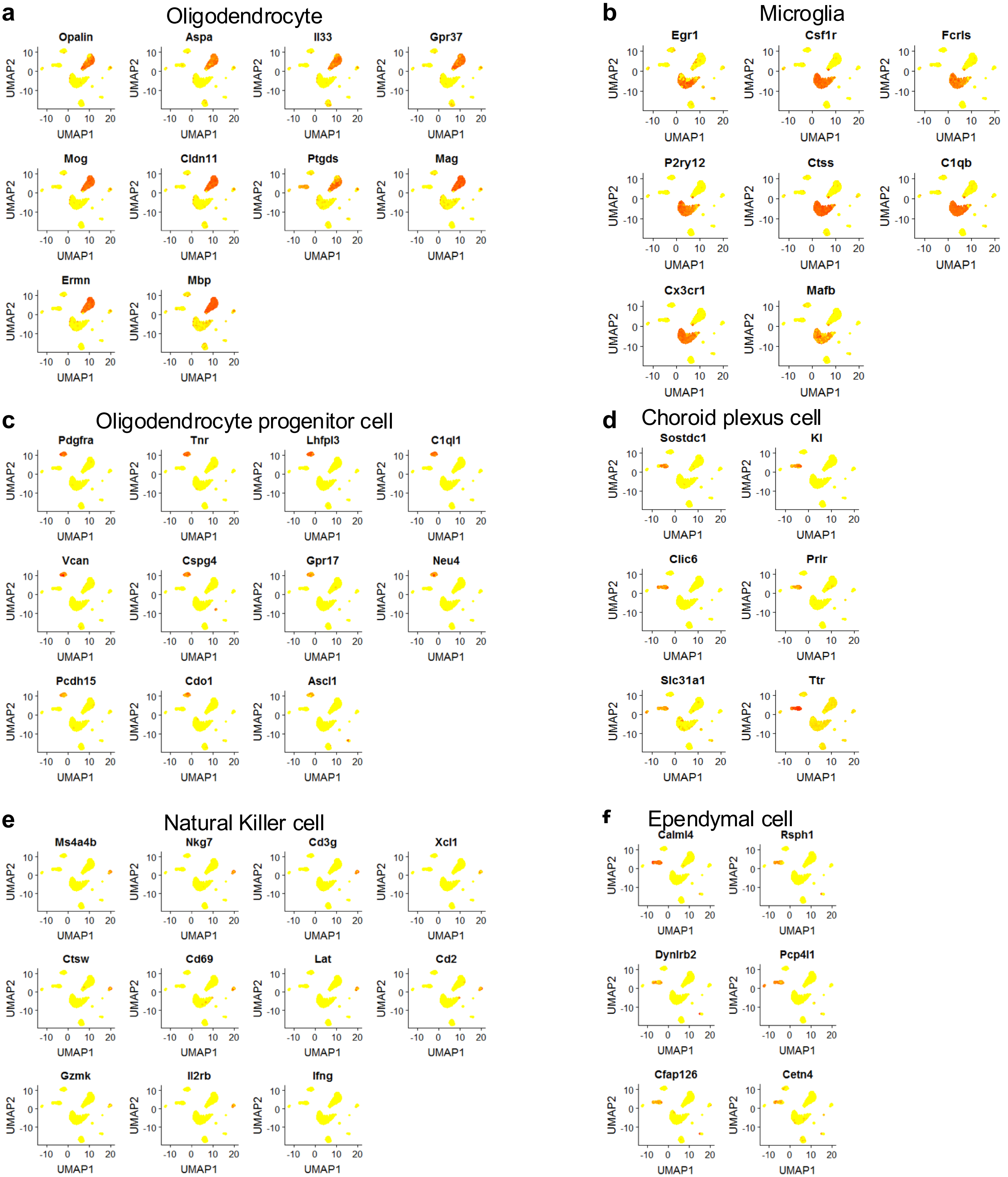

Supplemental Figure 1. Markers used for cell type identification. (A-F) Control hippocampal UMAP graphs depicting expression of selected oligodendrocyte (A), microglia (B), oligodendrocyte progenitor (C), choroid plexus (D), natural killer (E), and ependymal (f) cell markers.

Figure S2

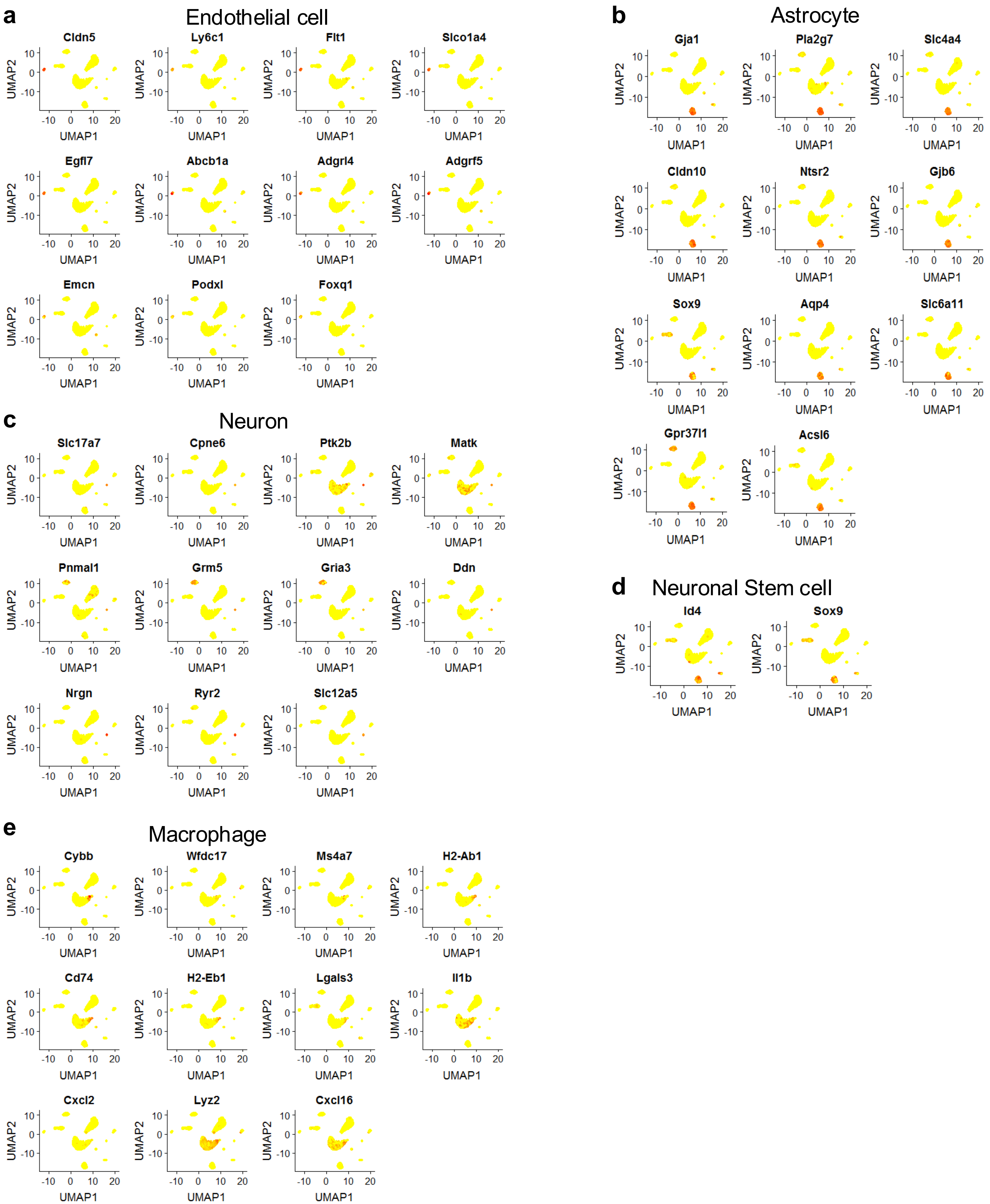

Supplemental Figure 2. Markers used for cell type identification. (A-E) Control hippocampal UMAP graphs depicting expression of selected endothelial (A), astrocyte (B), neuron (C), neural stem (D), and macrophage (E) cell markers.

Figure S3

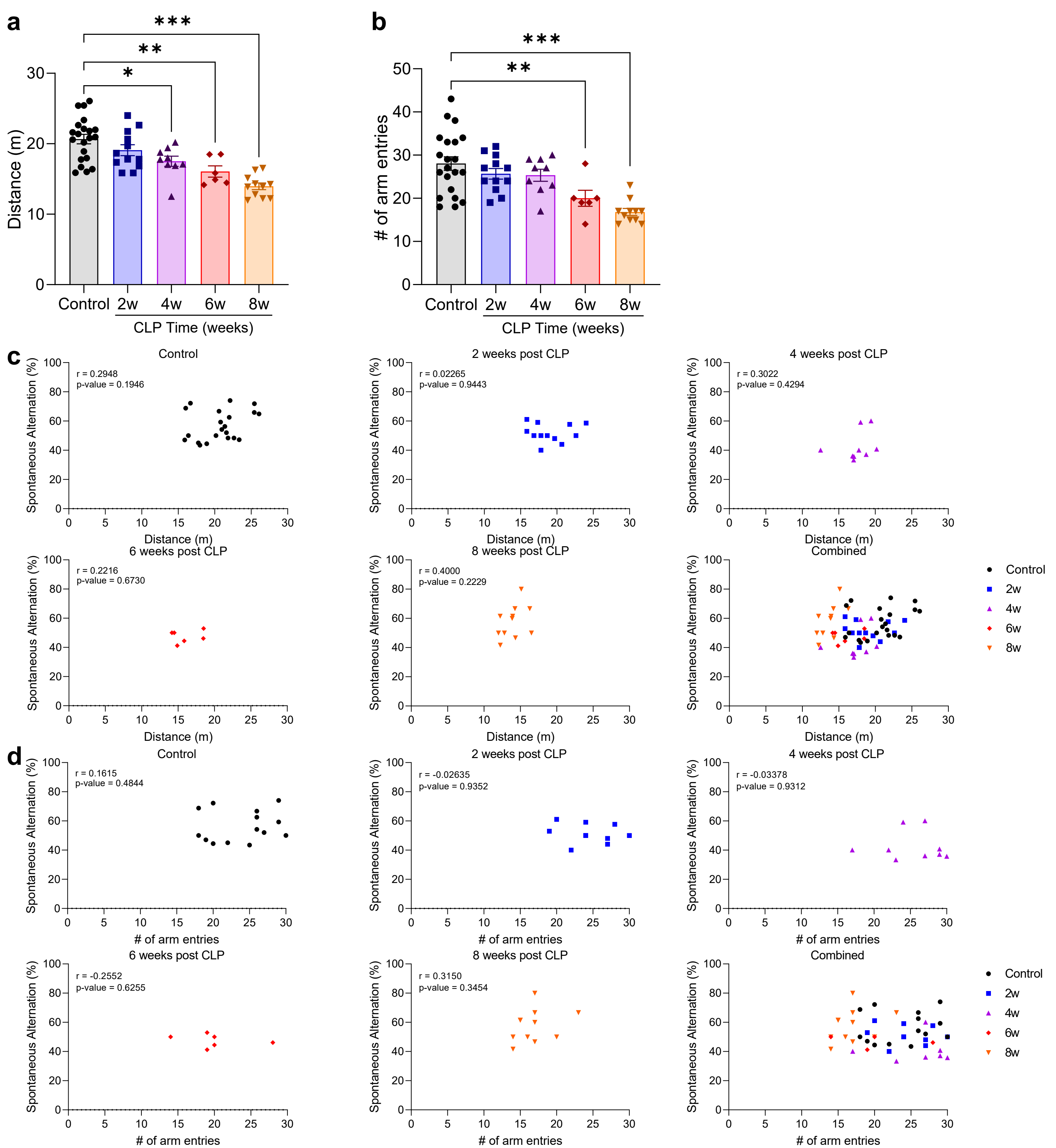

Supplemental Figure 3. Spontaneous alternation changes are not correlated with distance travelled or arm entries. (A-B) Quantification of distance travelled (A) and number of arm entries (B) from Y-maze in control and CLP mice at 2, 4, 6, and 8 weeks. (C-D) Pearson correlation graphs depicting distance (C) or number of arm entries (D) versus spontaneous alternation. A – B = One-way ANOVA with Bonferroni multiple comparisons (BMC). Data is represented as mean ± SEM. Significance: *p<0.05, **p<0.01, ***p<0.001.C – D = Pearson's correlation where significance is p<0.05. A - D, n = 6-21.

Figure S4

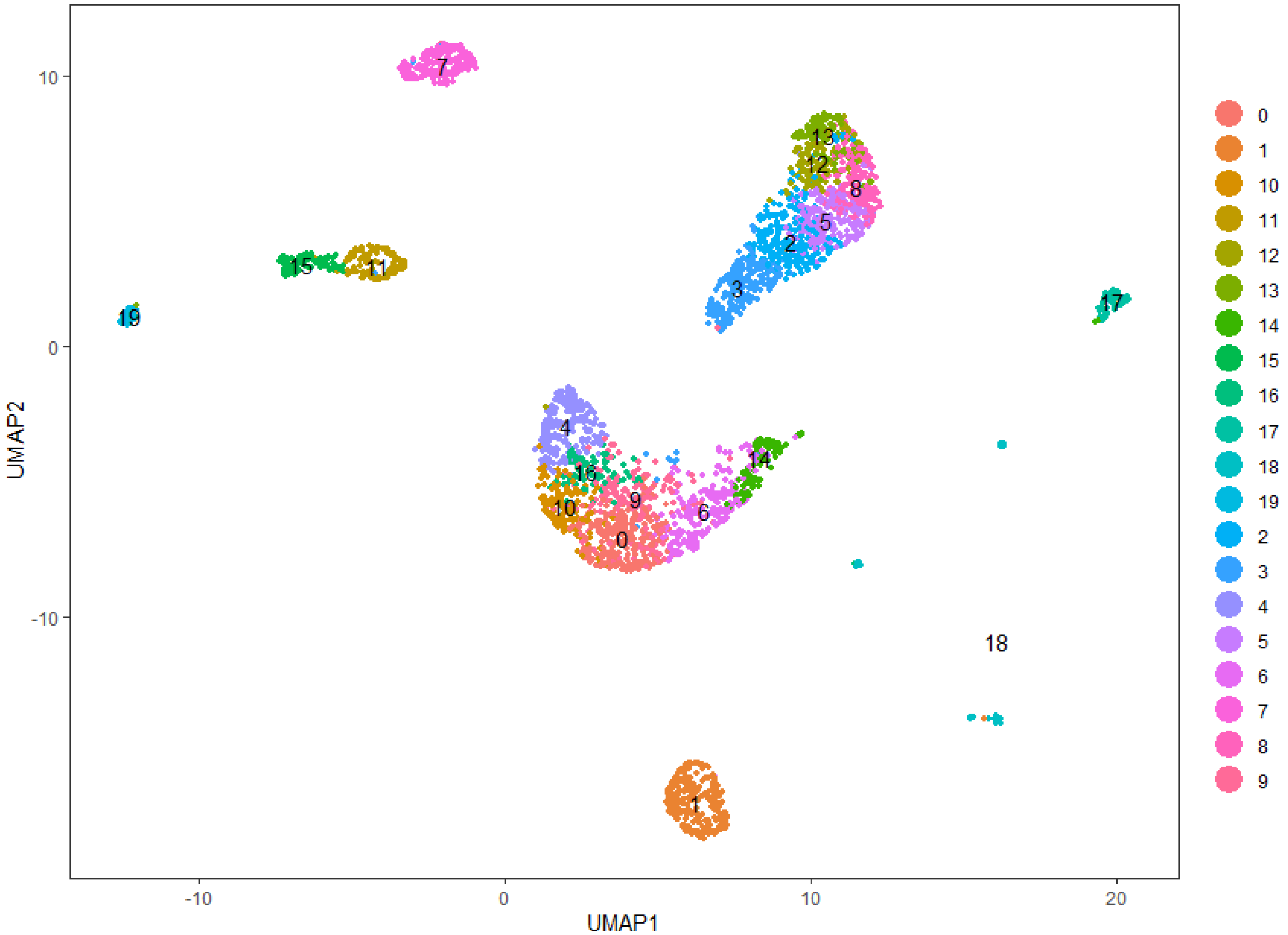

Supplemental Figure 4. Control hippocampus UMAP clustering. Control hippocampi cells separated into 20 cell clusters during single-cell analysis and were subsequently used for cell cluster identification in Figure 2A.
